# Sustainable energy policies from a complexity perspective

**DOI:** 10.3389/fdata.2023.1114796

**Published:** 2023-05-05

**Authors:** Rubén Alexander Acevedo-Rueda, Rodrigo Ramírez-Pisco, Carmen Luisa Vásquez Stanescu, Ennodio José Torres Cruz, Melva Inés Gómez-Caicedo, Mercedes Gaitán-Angulo

**Affiliations:** ^1^Doctorado en Ciencias de la Ingeniería mención Productividad, Universidad Nacional Experimental Politécnica Antonio José de Sucre, Barquisimeto, Venezuela; ^2^Universitat Carlemany, Sant Julià de Lòria, Andorra; ^3^Facultad de Ciencias Económicas, Administrativas y Contables, Fundación Universitaria los Libertadores, Bogotá, Colombia

**Keywords:** energy, sustainability, uncertainty models, complexity, model

## Abstract

The energy policies of the countries have become a key aspect of development. They must be formulated to guarantee economic and social development, state security and compliance with the objectives of sustainable development. In this framework, generation technologies must be considered not only in terms of available natural resources but also in terms of possible contingency scenarios. The purpose of this article is to prioritize technologies by applying a fuzzy inference model and uncertainty model and to address the principles of complex thinking to a case study. The methodology considers the integral vision of the dimensions under the systemic, feedback, autonomy/dependence, holographic and recursive principles, the assignment of weights for the dimension of sustainable development and, finally, the formulation of contingent scenarios. These scenarios consider: exhaustion of a primary source and change of technology with negative or positive impact. As a result, priority is given to the development of wind technology among renewable sources, followed by hydropower and geothermal. In the field of conventional energy, natural gas remains in the first place, since it also reinforces the security and fairness of the system. It is concluded that the process of formulating energy policies based on economic variables and the incorporation of sustainability, in terms of restrictions and linearity in the study models. This must be complemented with the adaptation of the legal and institutional framework that allows the fulfillment of the objectives that are expected to be achieved. Finally, it is necessary to keep constantly updated on changes and improvements in technology, which can modify the variables under study, in order to adapt strategies to new conditions.

## 1. Introduction

According to DeTombe and van Dijkum (2014), energy is of fundamental importance for humanity, considering that access to energy has become one of the main challenges to guarantee the development and security of the state, the eradication of poverty, social and economic transformation (Mulugetta et al., 2019; Wahlund and Palm, 2022). The formulation of policies in this area is aimed at supporting the sustainable development of countries and regions and is a process that merits a detailed analysis regarding in terms the applicable tools and models, as well as their particularities. According to OLADE (1997), these should be based mainly on temporary objectives rather than actions. These actions should be flexible as knowledge of your applications deepens and should be based on feedback.

The fight against poverty (Guzowski et al., 2021), social transformations and economic development (Ordeñana et al., 2022; Vardar et al., 2022), the needs of modern society for a quality energy supply (Mercado-Bautista et al., 2022; Wahlund and Palm, 2022; Wierzbicka, 2022) and guarantee the security of the state (Gaspar, 2022; Steffen and Patt, 2022) and, finally, move toward low-carbon generation sources (Lawrence et al., 2022), among other reasons, to frame what today is considered as energy policies sustainable (Nilsson, 2005).

According to the World Energy Council (WEC, 2011) energy sustainability is defined in three (3) dimensions:

- **Energy security**. For both importers and net exporters of energy, it includes effective management of primary energy supply from internal and external resources; the reliability of the energy infrastructure; and the ability of participating energy companies to meet current and future demands. In countries that are net exporters of energy, it also refers to the ability to maintain income from sales to foreign markets.
- **Social equity**. It refers to the accessibility and affordability of energy supply for the population.
- **Reducing of environmental impact**. It means efficiency in energy supply and demand, as well as in the development of energy supply from renewable resources and other low-carbon sources.

One of the problems presented by the concept of sustainable development is the measurement of compliance with its dimensions, an issue that has been the subject of studies and proposals, such as the one proposed by Bluszcz (2016). This author points out that its multidimensionality makes it difficult to accurately and exhaustively measure its social, economic and environmental components. This makes it necessary to use composite indicators (also called synthetic), which allow the aggregation of individual indicators with different units of measurement and even of a qualitative nature.

The indicators used by WEC (2011) are classified into the three (3) Human/Social, Ecological/Environmental and Economic dimensions and, within these, are grouped in turn by subcategory: Basic Needs, Personal Development and Health, Social Equity, Natural Resources, Climate and Energy and, finally, Transition and Economy.

Szopik-Depczyńska et al. (2018) point out that innovation initiatives within the framework of the indicators presented in the UN 2030 agenda have motivated companies to make investments in new technologies or modernization of existing assets, which could result in a decrease in energy expenditure and use of natural resources. In this sense, energy policy makers are forced to collaborate with their colleagues in other areas and governance structures are established to activate and maintain such coordination (Nerini et al., 2018).

It is proposed that in order to understand the application of the principles of complex thinking, proposed by Edgar Morin, to the formulation of energy policies, it is necessary to approach the study from the principles: Subject/Object, Systemic, Feedback, Autonomy/Dependence, Recursivity, Holographic, Uncertainty, Fuzzy, Situational, and Chaordic Strategy (Acevedo Rueda et al., 2020). The purpose of this article is to establish the prioritization of technologies by applying an uncertainty and fuzzy inference model that addresses the principles of complex thinking to a case study.

## 2. Sustainable energy policies

Energy systems are strongly identified and related to the different spheres of economic and environmental development. The ECLAC (2003) highlights the following considerations:

- Energy is an essential element for the quality of life of human beings; it is a widely used input in all productive activities.
- The availability of energy has played a central role in the process of human development.
- The great technological revolutions, which affected production and consumption activities, are intimately linked to the substitution between primary energy sources. Energy production and consumption have strong interactions with the natural environment. In addition to the possibility of depletion or degradation of energy resources, there are multiple negative impacts on the soil and, ultimately, on the water and the aerial environment, derived from production, transformation and use.

Sustainable energy is understood as the provision of an affordable, accessible and reliable service that meets economic and social needs, with attention to environmental aspects. It is a broad context that encompasses resource consumption, existing energy infrastructure, and development needs (Oxilia and Blanco, 2016).

Energy sustainability indicators have been addressed by the World Energy Council (WEC), applying a methodology called Energy Trilemma, which considers 32 indicators in each of the dimensions. In addition, it is also presented as a tool to support the formulation of energy policies (Imio and Fonseca-Prieto, 2022).

From a theoretical perspective, energy sustainability is the capacity of an organization or political community (State or Community of States) to cover the energy demand of its society, without affecting its environment to such an extent that it could break the continuity of this capacity in the future. The theoretical construction of this capacity complies with the principles of complex thought. On the other hand, from a methodological perspective, it is a measure that assigns a real value to the resulting set of variables considered for the problem in the Economic, Human/Social, Ecological/Environmental and Political/Institutional dimensions. Finally, from a theoretical-methodological perspective, it is the measure of capacity, which shows the expected value for a particular event, this event is a discrete condition, in a specific time horizon or instant, resulting from the combination of variables considered in the process under study, as an effect of the decisions (causes) assumed. The event of interest is the condition in which the system is sustainable, vs. the unsustainable condition.

## 3. Materials and methods

Fuzzy logic has found a place in different disciplines as a tool to handle the complexity of human language and sensory perception. It is common in decision-making processes or modeling and control of systems that present difficulties in their representation in classical logic (Klir and Yuan, 1995) and as a tool for decision-making under conditions of uncertainty (Ayyub, 2005). In addition, it has been applied in the control and optimization of variables associated with renewable energy systems, obtaining realistic estimates that justify the complexity of this approach, which moves away from the approximations and linearizations of traditional methods (Suganthi et al., 2015).

**Figure 1:**
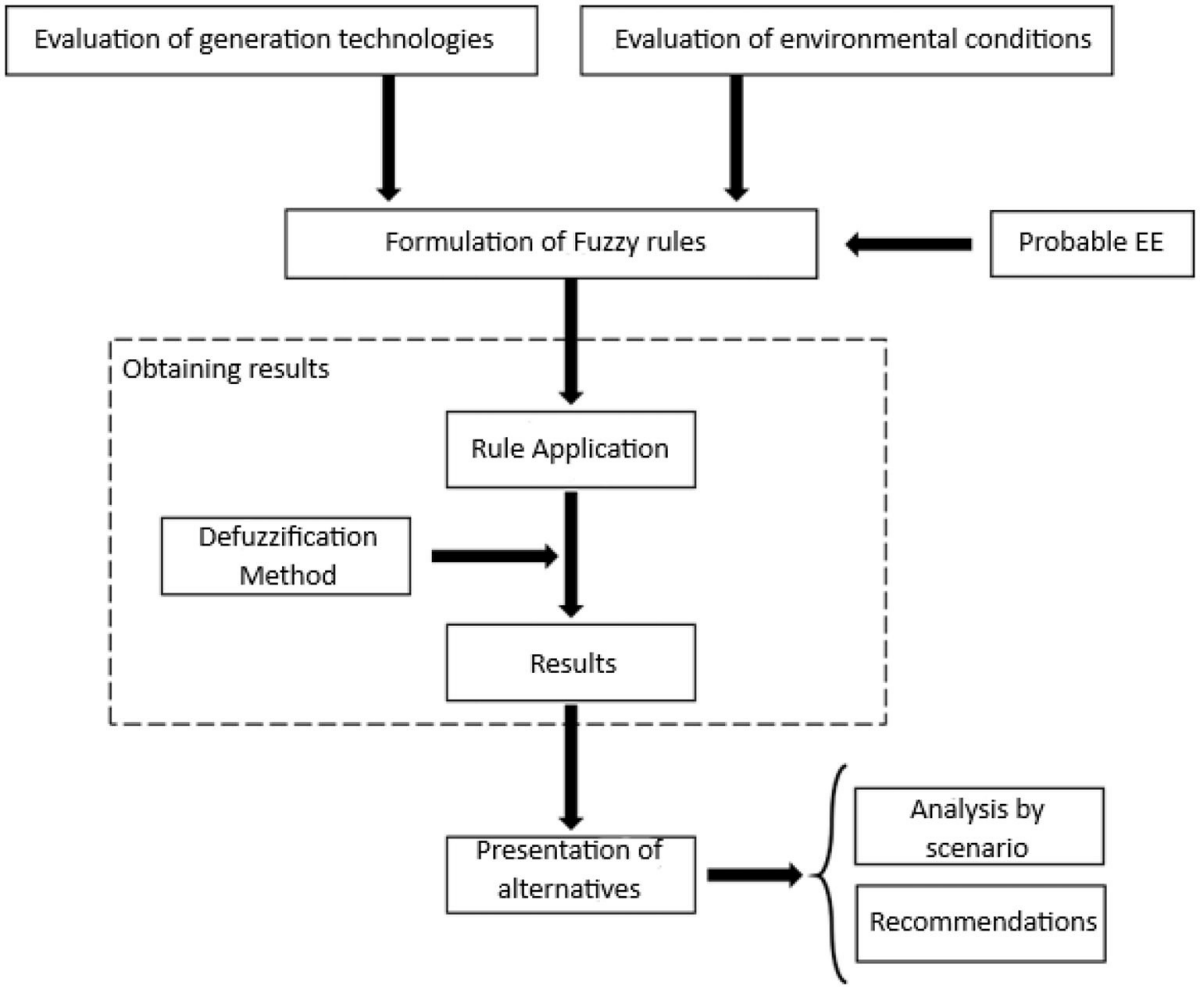
Methodology used to apply the principles of complex thinking. Source: Self made.

Figure 1 describes the proposed methodology for the application of the model considering the principles of uncertainty and fuzzy. For this, the principles of complex thinking are incorporated (Acevedo Rueda et al., 2020) and the following considerations were considered:

- **Assignment of weighted weights for each dimension of sustainable development**. Based on the indicators proposed by the Energy Trilemma (WEC, 2019) and their weightings, the subject/object principle is addressed by assigning a contribution from each of the subjects in the decision-making process.
- **Comprehensive vision of all dimensions**. The model contemplates decision making based on the relational evaluation of indicators of all dimensions of sustainable development, considering the impact that each of the decision alternatives has on the global indicators over time, as well as the impact of the current environmental conditions on the evaluation of alternatives. In this way, the principles of systemic, feedback, autonomy/dependence, holography and recursion are addressed.
- **Formulation of contingent scenarios**. The principles of uncertainty, situational and chaordic strategy are incorporated with the formulation of probable, possible and desirable scenarios to make decisions and establish contingency plans, considering the possibility of generating Fuzzy Energy Events (FEE), typified as Creative Fuzzy Energy Events (C) or Destructive Fuzzy Energy Events (D).

For the formulation of the contingent scenarios, the methodology described below is used:

1. Evaluation of generation technologies:

a. Evaluation of impact indicators in the dimensions of sustainable development.
b. Identification of the linguistic scale for the weighting of the criteria.
c. Construction of the comparative matrix and fusion of the criteria.

2. Evaluation of environmental conditions:

a. Evaluation of the indicators present in the dimensions of sustainable development.
b. Identification of the linguistic scale for the weighting of the criteria.
c. Construction of the comparative matrix and fusion of the criteria.
d. Identification of risks in environmental conditions.

3. Formulation of fuzzy rules:

a. Analysis of the relationships between variables.
b. Identification of the linguistic scale for the weight of relationships.
c. Incorporation of probable EEDs.

4. Obtaining the results:

a. Selection of the defuzzification method.
b. Application of fuzzy rules for each probable EED (contingent scenario).
c. Defuzzification of results.

5. Presentation of alternatives:

a. Analysis of each contingent scenario with identification of EEDsC and EEDsD.
b. Formulation of recommendations for the follow-up and monitoring of results.

## 4. Description of the analysis variables

**Figure 2:**
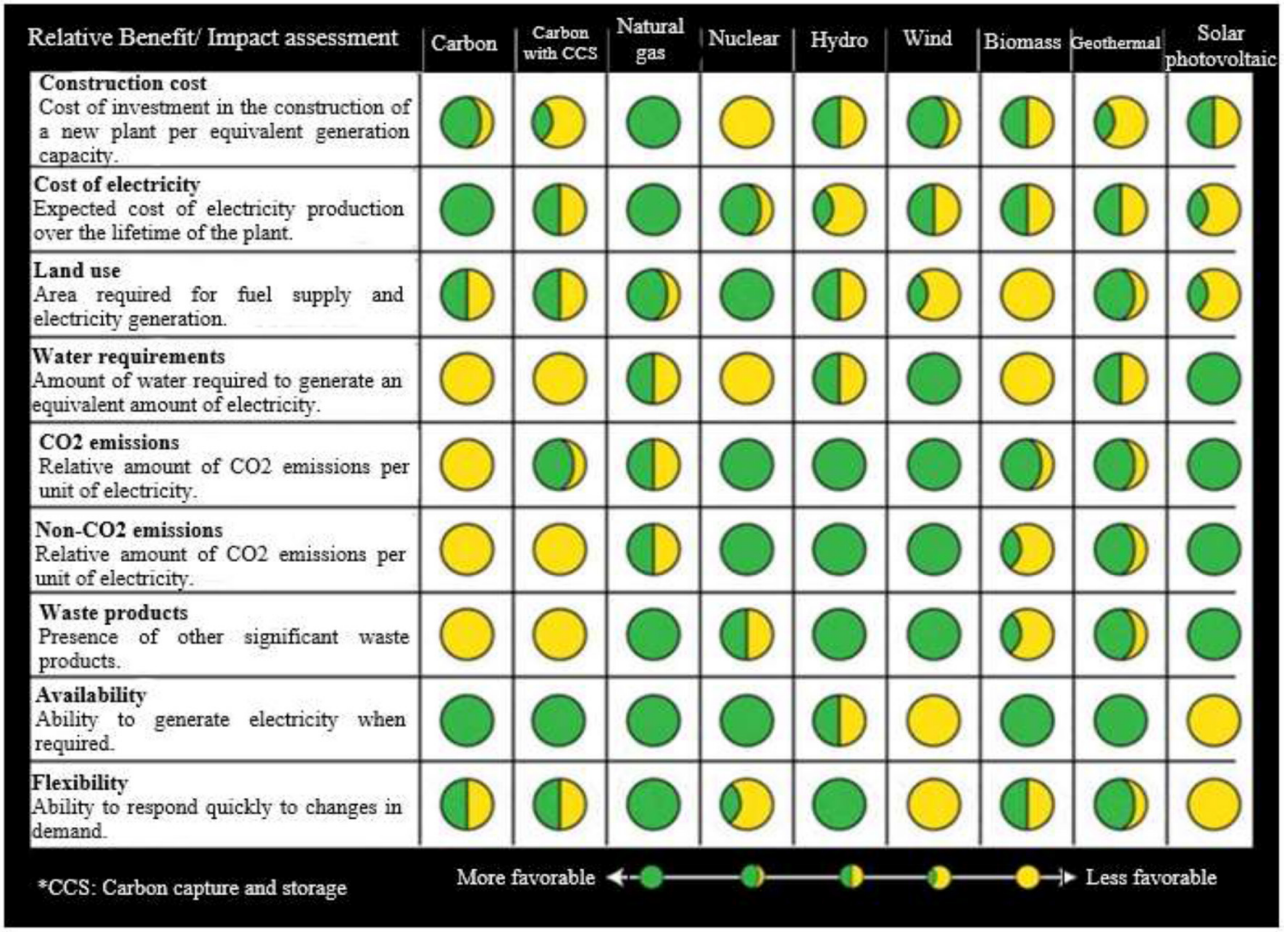
EPRI reference table of generation technologies (Carlberg, 2013).

Decision making in the formulation of energy policies from the perspective of complexity requires evaluating the impact of the alternatives on the selected indicators. Carlberg (2013) presents the rating system of the Electric Power Research Institute (EPRI) which develops a reference table for the evaluation of generation technologies, including renewables, natural gas, coal and nuclear, in terms of their relative impact on specific areas, as presented in Figure 2.

In this aspect, the available electricity generation technologies are considered, with the characteristics developed in the literature (IRENA, 2018, 2020; CNE, 2020; NREL, 2020). The values are weighted according to the maximums in each variable and, based on what is presented in Figure 2, the values not available in the references are estimated, under the following considerations:

- The generation technologies evaluated are Coal, Diesel, Natural Gas, Hydraulic, Wind, Biomass, Geothermal and Solar Photovoltaic.
- The Emissions variable is defined, encompassing CO_2_ and non-CO_2_.
- The variables take values proportional to their maximum, between 0.01 and 1.00. Where 1.00 represents the maximum value in the set of technologies evaluated and the others correspond to the proportion with respect to this.

Table 1 presents the weighted variables for the classification of power generation technologies in the proposed fuzzy model.

**Table 1:** Variables for the fuzzy model for classifying power generation technologies.

		**Technology**							
**Variable**	**Description**	**Coal**	**Diesel**	**Natural**	**Hydro**	**Wind**	**Biomass**	**Geothermal**	**Solar photovoltaic**
I1	Construction cost	0.48	0.35	0.28	1.00	0.22	0.56	0.71	0.68
I2	Cost of electricity	0.37	1.00	0.55	0.35	0.20	0.82	0.46	0.25
I3	Land use	0.50	0.10	0.10	0.50	0.50	1.00	0.40	0.85
I4	Water requirements	1.00	1.00	0.50	0.50	0.10	1.00	0.65	0.10
I5	Pollution	1.00	1.00	0.50	0.10	0.10	0.30	0.30	0.10
I6	Availability	0.80	0.80	0.90	0.65	0.30	0.75	0.75	0.25
I7	Flexibility	0.50	0.70	0.90	0.90	0.15	0.50	0.20	0.15

Source: Own elaboration with information from Carlberg (2013), IRENA (2018), CNE (2020), IRENA (2020), and NREL (2020).

For decision making, the impact of the alternatives on the selected indicators will be evaluated and the development of installations of these technologies will be prioritized.

### 4.1. Evaluation of environmental conditions

For the evaluation variables of the environmental conditions, the rating of the energy trilemma index is taken, in each of its dimensions. This has a percentage weighting that can also be projected to values between zero (0) and one (1), with zero (0) corresponding to 0% and one (1) to 100%. The variable installed capacity by technology is included in these dimensions, to incorporate the diversity of the energy resource in the analysis, with values between zero (0) and one (1). Where zero (0) indicates that there is no installed capacity of a certain technology and one (1) represents the use of a single technology to cover the total energy requirement. The variables considered for the evaluation of environmental conditions in the fuzzy model are presented in Table 2.

**Table 2:** Environmental conditions assessment variables for the fuzzy model.

**Variable**	**Description**
C1	Energy security
C2	Energy equity
C3	Environmental sustainability of energy systems
C4	Installed capacity by technology

### 4.2. Variable fusion

Both the variables that represent the impact of each alternative and the environment variables were characterized in the set [0,1] and the membership functions are modeled considering the sets Low (B), Medium (M), and High (A), so:

Membership function to the set Under μ_*B*_(*x*) of type L, function Equation (1).


(1) $$\begin{array}{l} \mu_{B}\left(x\right)=\max\left(min\left(\frac{b-x}{b-a},0\right),1\right) \end{array}$$

Set membership function of the set Medium μ_*M*_(*x*) of type Triangular Equation (2).


(2) $$\begin{array}{l} \mu_{M}\left(x\right)=\max\left\{min\left(\frac{x-a}{b-a},\frac{c-x}{c-b}\right),0\right\} \end{array}$$

High set membership function μ_*A*_(*x*) of type Gamma Linear Equation (3).


(3) $$\begin{array}{l} \mu_{A}\left(x\right)=\max\left(min\left(\frac{x-b}{c-b},0\right),1\right) \end{array}$$

For the variable C1, corresponding to the security condition of the energy trilemma index, given that the results presented for the group of qualified countries are between 30 and 79%, the following is assigned (*a*; *b*; *c*) = (0.50; 0.60; 0.65). Thus, the membership functions are defined as Equations (4)–(6).


(4) $$\begin{array}{lll} \mu_{B}\left(x\right) & = & \max\left(min\left(\frac{0.60-x}{0.10},0\right),1\right) \end{array}$$

(5) $$\begin{array}{lll} \mu_{M}\left(x\right) & = & \max\left\{min\left(\frac{x-0.50}{0.10},\frac{0.65-x}{0.05}\right),0\right\} \end{array}$$

(6) $$\begin{array}{lll} \mu_{A}\left(x\right) & = & \max\left(min\left(\frac{x-0.60}{0.05},0\right),1\right) \end{array}$$

For the other variables, we assign (*a*; *b*; *c*) = (0.25; 0.5; 1) to the other variables, distributing the range of values evenly. Thus, the membership functions are defined as Equations (7)–(9).


(7) $$\begin{array}{lll} \mu_{B}\left(x\right) & = & \max\left(min\left(\frac{0.25-x}{0.25},0\right),1\right) \end{array}$$

(8) $$\begin{array}{lll} \mu_{M}\left(x\right) & = & \max\left\{min\left(\frac{x-0.25}{0.25},\frac{0.75-x}{0.25}\right),0\right\} \end{array}$$

(9) $$\begin{array}{lll} \mu_{A}\left(x\right) & = & \max\left(min\left(\frac{x-0.5}{0.25},0\right),1\right) \end{array}$$

To reduce the effect of conscious ignorance due to imprecision (Ayyub, 2005) further fine- tuning the output with the sets Very Low (MB), Low (B), Medium (M), High (A), Very High (MA), as follows:

- Very Low set membership function μ_B_(*x*) of type L Equation (10).

(10) $$\begin{array}{l} \mu_{MB}\left(x\right)=\max\left(min\left(\frac{b-x}{b-a},0\right),1\right) \end{array}$$

- Set membership function Low μ_A_(*x*) of type Triangular Equation (11)

(11) $$\begin{array}{l} \mu_{B}\left(x\right)=\max\left\{min\left(\frac{x-a}{b-a},\frac{c-x}{c-b}\right),0\right\} \end{array}$$

- Membership function of the set Medium μ_*M*_(*x*) of type Triangular Equation (12).

(12) $$\begin{array}{l} \mu_{M}\left(x\right)=\max\left\{min\left(\frac{x-b}{c-d},\frac{d-x}{d-c}\right),0\right\} \end{array}$$

- Very High set membership function μ_*A*_(*x*) of Triangular type Equation (13).

(13) $$\begin{array}{l} \mu_{MA}\left(x\right)=\max\left\{min\left(\frac{x-c}{d-c},\frac{e-x}{e-d}\right),0\right\} \end{array}$$

High set membership function μ_*A*_(*x*) of type Gamma Linear Equation (14).


(14) $$\begin{array}{l} \mu_{A}\left(x\right)=\max\left(min\left(\frac{x-d}{e-d},0\right),1\right) \end{array}$$

It is assigned (*a*; *b*; *c*; *d*; *e*) = (0.19; 0.38; 0.57; 0.76; 0.95) to evenly distribute the range of values. Thus, the membership functions are defined as Equations (15)–(19).


(15) $$\begin{array}{lll} \mu_{MB}\left(x\right) & = & \max\left(min\left(\frac{0.38-x}{0.19},0\right),1\right) \end{array}$$

(16) $$\begin{array}{lll} \mu_{B}\left(x\right) & = & \max\left\{min\left(\frac{x-0.19}{0.19},\frac{0.57-x}{0.19}\right),0\right\} \end{array}$$

(17) $$\begin{array}{lll} \mu_{M}\left(x\right) & = & \max\left\{min\left(\frac{x-0.38}{0.19},\frac{0.76-x}{0.19}\right),0\right\} \end{array}$$

(18) $$\begin{array}{lll} \mu_{MA}\left(x\right) & = & \max\left\{min\left(\frac{x-0.57}{0.19},\frac{0.95-x}{0.19}\right),0\right\} \end{array}$$

(19) $$\begin{array}{lll} \mu_{A}\left(x\right) & = & \max\left(min\left(\frac{x-0.76}{0.19},0\right),1\right) \end{array}$$

**Figure 3:**
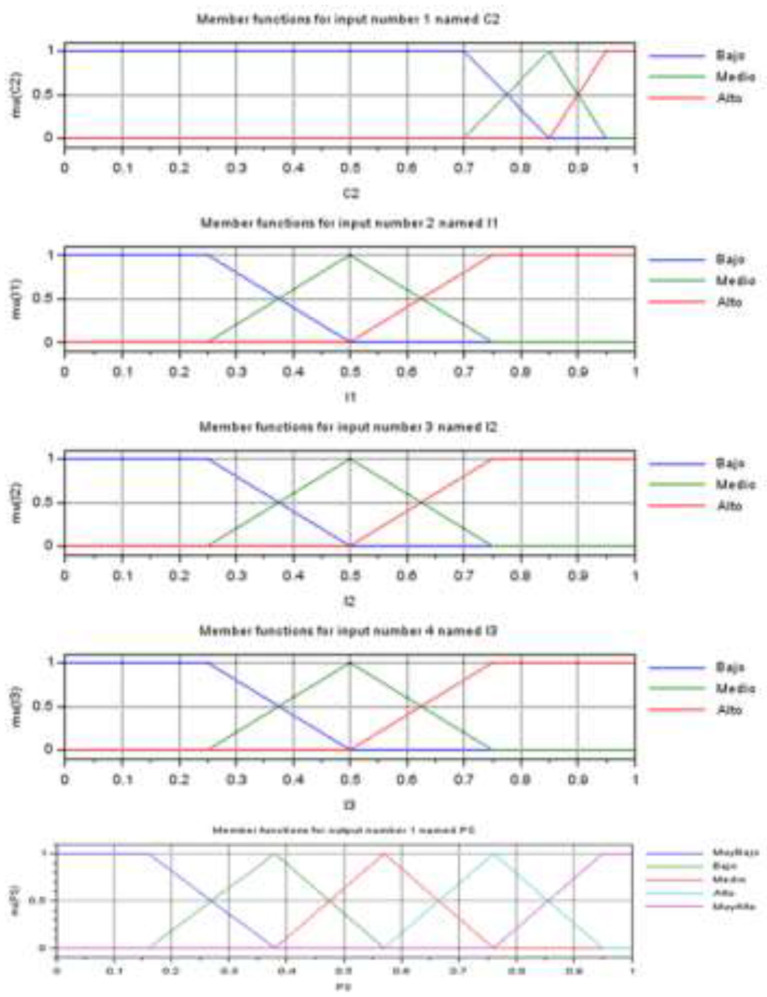
Belonging functions.

Figure 3 shows the graphs of membership functions for a set of variables.

### 4.3. Formulation of fuzzy rules

**Figure 4:**
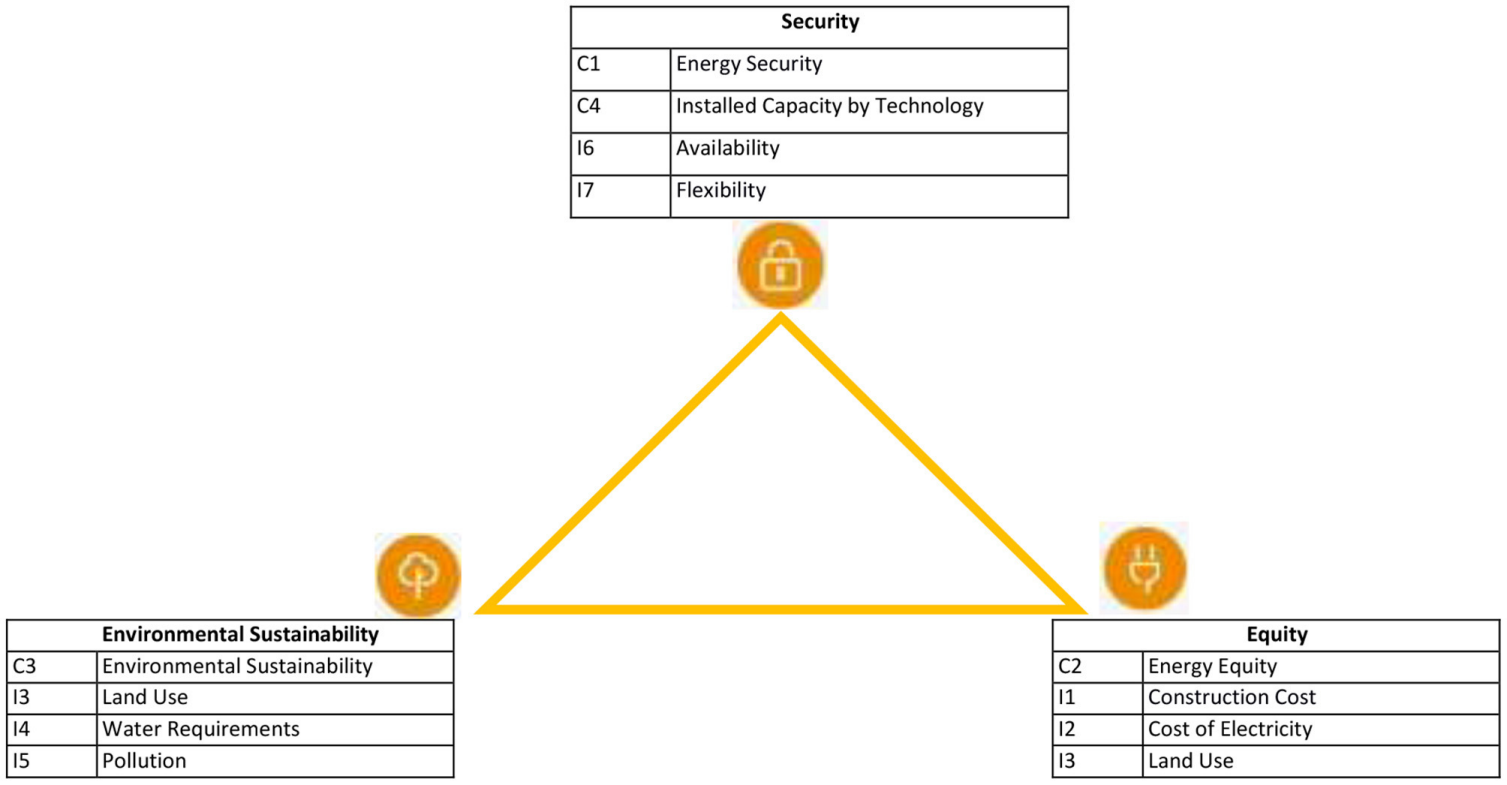
Ranking of variables by impact on the energy sustainability index.

To formulate the fuzzy rules, it is necessary to identify and classify the variables according to their impact on the energy sustainability index. In this way, the benefit offered by each alternative on the corresponding dimensions can be obtained as a result, to finally obtain a categorization of the technologies evaluated under the existing environmental conditions. This benefit is also set in a range from zero (0) to one (1) and is modeled considering the sets Low (B), Medium (M), High (A), with the same parameters and membership functions used for the model variables. Figure 4 presents the classification of the variables according to their impact on the energy trilemma index.

By incorporating the rating of the energy trilemma index as environmental conditions, fuzzy rules allow higher priority to be given to technologies that offer greater benefits to the dimensions that require it most. As a rule of thumb, they are formulated as follows:

**IF** [Environment Variable is (A, M, B)] **AND** [Variable is (A, M, B)] **THEN** Benefit is (MA, M, B, MB).

Where the notation (U is V) denotes the intersection of the conditions for the variables considered, i.e., [Variable is (A, M, B)] = I1 is A **AND** I2 is M **AND** I3 is B.

**Figure 5:**
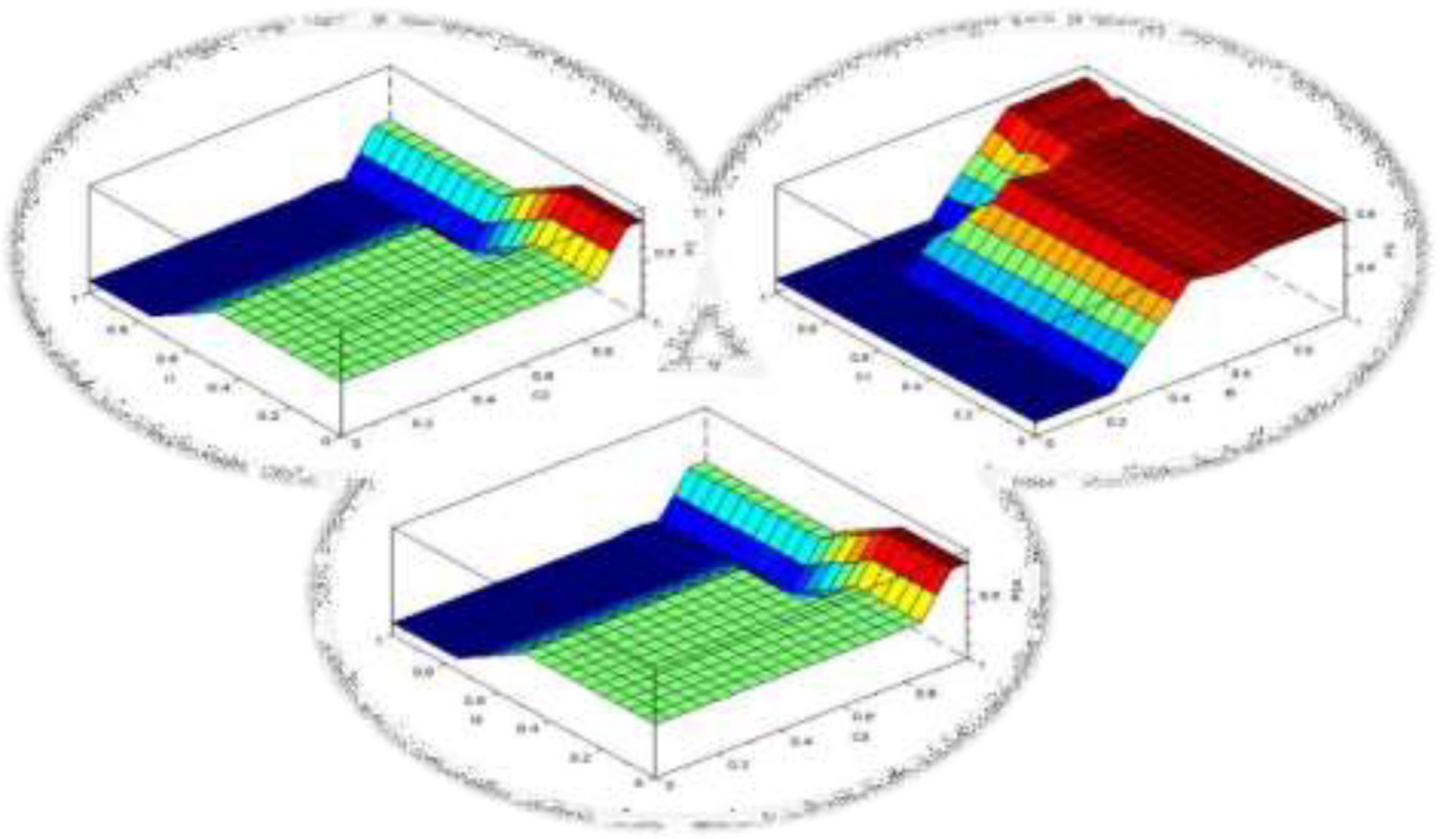
Fuzzy rule surfaces.

With this classification of the variables, a total of 243 rules are obtained, distributed in three groups of 81 rules each. Figure 5 shows a graphical representation of the fuzzy surfaces for three (3) variables in each domain.

The results obtained after defuzzification in each group will be added to obtain the final benefit of each evaluated technology. This provides valuable information for decision-making in the formulation of energy policies.

### 4.4. Probable diffuse energy events

To identify likely diffuse Energy Events, one must maintain an expert understanding of what variable relationship factors or perturbations can change, which can alter the dynamics of the system. For this work, events with the following characteristics will be considered in particular:

- Depletion of a primary energy source. This can be caused by technical, legal, political or environmental restrictions that prevent the exploitation of a certain resource. In the case of fuel-importing countries, it could be due to a breakdown in relations with suppliers; in other cases, it could be due to the inability to meet water or land use requirements.
- Negative change in the impact of a technology. This can be caused by technical, legal, political or environmental conditions that increase the costs of exploiting a certain resource, such as taxes or goods.
- Technological improvements that change the relationship in one or more variables, with positive impact.

In this phase, the alternatives for the base case and the cases of the contingent scenario are evaluated, corresponding to the occurrence of the probable EEDs identified.

## 5. Results

In this phase, the alternatives for the base case and the cases of the contingent scenario are evaluated, corresponding to the occurrence of the probable EEDs identified, and the centroid method is used to defuzzify the result. The result presents a score for each technology in the dimensions of safety, equity and environmental sustainability, which are then consolidated as a summation into a single indicator.

As a test example, we work with a hypothetical case that presents a result of 68, 61, and 48% in safety, equity and environmental sustainability, respectively, and an installed capacity per technology, as shown in Table 3. In addition, primary sources are assumed to be available for all technologies under evaluation.

**Table 3:** Categorization of installed capacity by technology.

**Coal**	**Diesel**	**Natural gas**	**Hydro**	**Wind**	**Biomass**	**Geothermal**	**Solar photovoltaic**
0.70	0.65	1.00	0.95	0.25	0.10	0.10	0.15

### 5.1. Base case

Applying the fuzzy model to these values yields the results presented in Table 4.

**Table 4:** Priorities base case.

**Technology**	**Security**	**Equity**	**Sust. medioamb**.	**Total**	**Priority**
Coal	0.414	0.668	0.140	1.222	
Diesel	0.594	0.369	0.140	1.102	8
Natural gas	0.570	0.647	0.760	1.977	1
Hydro	0.479	0.479	0.760	1.719	
Wind	0.191	0.760	0.808	1.759	
Biomass	0.760	0.150	0.325	1.234	
Geothermal	0.570	0.491	0.636	1.697	
Solar photovoltaic	0.140	0.428	0.760	1.328	5

### 5.2. Contingent scenario 1. Primary source depletion

When considering the EED of depletion of primary sources, the technologies that require it are eliminated. The prioritization among the remaining alternatives is maintained. As a hypothetical case, the joint case of decarbonization of the energy matrix due to political provisions and the depletion of hydroelectric resources due to the lack of suitable spaces for the construction of new dams is considered. Under these assumptions, the prioritization would be as shown in Table 5.

**Table 5:** Priorities contingent scenario 1.

**Technology**	**Security**	**Equity**	**Sust. medioamb**.	**Total**	**Priority**
Coal	0.414	0.668	0.140	1.222	-
Diesel	0.594	0.369	0.140	1.102	-
Natural gas	0.570	0.647	0.760	1.977	1
Hydro	0.479	0.479	0.760	1.719	-
Wind	0.191	0.760	0.808	1.759	
Biomass	0.760	0.150	0.325	1.234	5
Geothermal	0.570	0.491	0.636	1.697	
Solar photovoltaic	0.140	0.428	0.760	1.328	

### 5.3. Contingent scenario 2. Negative change in the impact of an alternative

When considering the EED of negative change in the impact of an alternative, the values in the matrix of variables by technology are modified and the fuzzy model is applied again to obtain the prioritization for this contingent scenario. As a hypothetical case, the application of taxes on emissions and construction permits, and policies restricting the dispatch of non-renewable technologies, which modify the values as shown in Table 6, are considered. Under these assumptions, the prioritization would be as presented in Table 7.

**Table 6:** Variables for the fuzzy model contingent scenario 2.

		**Technology**							
**Variable**	**Description**	**Coal**	**Diesel**	**Natural gas**	**Hydro**	**Wind**	**Biomass**	**Geothermal**	**Solar photovoltaic**
I1	Construction cost	1.00	0.95	0.48	0.80	0.12	0.45	0.65	0.52
I2	Cost of electricity	0.45	1.00	0.65	0.35	0.20	0.85	0.46	0.25
I3	Land use	0.64	0.25	0.23	0.62	0.50	1.00	0.40	0.85
I4	Water requirements	1.00	1.00	0.64	0.54	0.10	1.00	0.65	0.10
I5	Pollution	1.00	1.00	0.58	0.10	0.10	0.45	0.30	0.10
I6	Availability	0.70	0.75	0.80	0.65	0.30	0.75	0.75	0.25
I7	Flexibility	0.50	0.70	0.90	0.90	0.15	0.50	0.20	0.15

Source: Own elaboration with information from Carlberg (2013); IRENA (2018, 2020); CNE (2020), and NREL (2020).

**Table 7:** Priorities contingent scenario 2.

**Technology**	**Security**	**Equity**	**Sust. medioamb**.	**Total**	**Priority**
Coal	0.370	0.420	0.159	0.949	
Diesel	0.594	0.140	0.140	0.873	8
Natural gas	0.570	0.531	0.462	1.563	
Hydro	0.479	0.467	0.619	1.565	
Wind	0.191	0.760	0.808	1.759	1
Biomass	0.760	0.148	0.191	1.098	
Geothermal	0.570	0.534	0.636	1.741	
Solar photovoltaic	0.140	0.545	0.760	1.445	5

### 5.4. Contingent scenario 3. Technological improvements

When considering the EED of technological improvements, the values of the matrix of variables by technology are modified in favor of an alternative and the fuzzy model is applied again to obtain the prioritization for this contingent scenario. As a hypothetical case, the case of photovoltaic solar technology is considered, with the incorporation of storage equipment that improves availability, improvements in inverters and panels, which increase flexibility, reduce construction costs and land use, which modifies the values as shown in Table 8. Under these assumptions, the prioritization would be as shown in Table 9.

**Table 8:** Variables for the fuzzy model contingent scenario 3.

		**Technology**							
**Variable**	**Description**	**Coal**	**Diesel**	**Natural gas**	**Hydro**	**Wind**	**Biomass**	**Geothermal**	**Solar photovoltaic**
I1	Construction cost	0.48	0.35	0.28	1.00	0.22	0.56	0.71	0.35
I2	Cost of electricity	0.37	1.00	0.55	0.35	0.20	0.82	0.46	0.25
I3	Land use	0.50	0.10	0.10	0.50	0.50	1.00	0.40	0.60
I4	Water requirements	1.00	1.00	0.50	0.50	0.10	1.00	0.65	0.10
I5	Pollution	1.00	1.00	0.50	0.10	0.10	0.30	0.30	0.10
I6	Availability	0.80	0.80	0.90	0.65	0.30	0.75	0.75	0.45
I7	Flexibility	0.50	0.70	0.90	0.90	0.15	0.50	0.20	0.25

Source: Own elaboration with information from Carlberg (2013), IRENA (2018), CNE (2020), IRENA (2020), and NREL (2020).

**Table 9:** Priorities contingency scenario 3.

**Technology**	**Security**	**Equity**	**Sust. medioamb**.	**Total**	**Priority**
Coal	0.414	0.668	0.140	1.222	
Diesel	0.594	0.369	0.140	1.102	8
Natural gas	0.570	0.647	0.760	1.977	1
Hydro	0.479	0.479	0.760	1.719	
Wind	0.191	0.760	0.808	1.759	
Biomass	0.760	0.150	0.325	1.234	
Geothermal	0.570	0.491	0.636	1.697	5
Solar photovoltaic	0.325	0.680	0.788	1.793	

## 6. Analysis of results

The results obtained show the priority given to the development of projects with wind technology in renewable energies, which remains in first place among these technologies in all scenarios, in addition to hydraulic and geothermal. Solar photovoltaic does not reach the first places, which is explained by the low availability and flexibility, in addition to the extensive use of land assigned in the evaluation, but in the scenario of improving of its technology, it reaches the first place in the renewable priorities. In the conventional energy category, natural gas remains in first place, while coal and diesel follow in second and third place, respectively.

Considering the availability of all primary resources for exploitation, in these scenarios the development of natural gas projects is recommended to strengthen security and equity. For the security indicator, biomass offers the best result, which is explained by its low presence in the energy matrix and the good level of availability and flexibility it offers. Wind and solar photovoltaic technology offer improvements in equity and environmental sustainability indicators, so their use is also considered favorable. It is necessary to keep constantly updated on changes and improvements in technology, which can modify the variables under study, in order to adapt strategies to new conditions.

The contingent scenarios, as analyzed, constitute CGEsC that allow for the diversification of the energy matrix, with the incorporation of priorities to non-conventional technologies. If contingent scenario 1 occurs as a total depletion, due to disasters or conflicts that completely interrupt the supply of the primary resource or cause the destruction of the existing infrastructure, it would be a CGED that would collapse the energy system, reinforcing the recommendation to develop technological projects that increase safety through the diversification of electricity generation.

This must be complemented with the revision of economic policies that favor investment in the technologies of interest, as well as the adaptation of the legal and institutional framework that allows the fulfillment of the objectives that are expected to be achieved. In this sense, the evaluation of the dimensions in the fuzzy model can include weights or incentives that adjust priorities.

## 7. Conclusions

This paper presents a model based on fuzzy logic to prioritize the development of electricity generation technologies, with a formulation of contingent scenarios, to address the principles of complex thinking, especially the principles of uncertainty and situational strategy, for a case study with availability of primary resources for the technologies considered: coal, diesel, natural gas, hydraulic, wind, biomass, geothermal and solar photovoltaic as the primary source of generation.

The problem was approached with a comprehensive vision of the dimensions under the systemic, feedback, autonomy/dependence, holographic and recursive principles; weights were assigned for the dimension of sustainable development, including 11 analysis variables based on the trilemma of electrical safety, energy, equity and environmental sustainability. Finally, contingent scenarios were formulated. These scenarios considered: exhaustion of a primary source and change technology with negative or positive impact.

As a result, wind power technology was found to be the most important renewable energy source in the case study, followed by hydropower and geothermal power. In the field of conventional energy, natural gas remains in the first place, since it also reinforces the security and fairness of the system.

It is concluded that the process of formulating energy policies based on economic variables and the incorporation of sustainability, in terms of restrictions and linearity in the study models. This must be complemented with the adaptation of the legal and institutional framework that allows the fulfillment of the objectives that are expected to be achieved. Finally, it is necessary to constantly update the model, based on changes and improvements in technology, which can modify the variables under study, strategies to the new conditions of the environment.

## Data availability statement

The original contributions presented in the study are included in the article/Supplementary material, further inquiries can be directed to the corresponding authors.

## Author contributions

All authors listed have made a substantial, direct, and intellectual contribution to the work and approved it for publication.
