# Supplementary material for: Sustainable energy policies from a complexity perspective
Source: Front Big Data. 2023 May 5;6:1114796. doi: 10.3389/fdata.2023.1114796 (PMC10204768; doi:10.3389/fdata.2023.1114796)
Supplement: Supplementary file 1 [file Data_Sheet_1.pdf]

**Step 1. Identification of the Chemical Substance**		
**Step 2. Assignment of BRQs in the 21 endpoints of the chemical under review**		
Green	No hazard identified for the criterion analysed	
Yellow	Limit hazard identified for the analysed criterion	
Grey	There is insufficient data available to determine the hazard level for the criterion under consideration.	
Red	Considered dangerous for the criterion analysed	
**Step 3. Assignment of the BRQ for Combined Aquatic Toxicity**		
**Fish toxicity**<br>**Toxicity of daphnia**<br>**Toxicity of algae**	→	**Aquatic Toxicity**
**Aquatic toxicity**<br>**Persistence**<br>**Bioaccumulation**	→	**Combined Aquatic Toxicity**
**Step 4. Exposure assessment for the chemical under review**		
If exposure is NOT plausible to humans or to AM via oral, dermal, or inhalation routes through volatile emissions, water, or other routes		
**Red**<br>**Grey**	→	**Yellow**
If exposure is plausible to humans or to AM via oral, dermal, or inhalation routes through volatile emissions, water, or other routes		
**Red**	→	**Red**
**Grey**	→	**Grey**
**Step 5. Chemical hazard qualification of the chemical under review.**		
a	Ideal safe chemical	
b	Chemical substance without moderate or significant risk	
c	Chemical with one (1) or more moderate hazards	
grey	Insufficient data to assess the chemical	
x	Chemical with one (1) or more significant hazards	
**Stage 6. RSH chemical risk rating**		
A	Healthy material	
B	Material that largely supports the C2C paradigm	
C	Material with some concerns	
GREY	Material cannot be fully assessed due to lack of data.	
X	Material with highly problematic properties, should be phased out	

FLAG	Material containing one or more chemicals as intentional inputs above 1000 ppm.

Figure 1. Stages of the eco-effective chemical risk assessment model at RSH.
