# Supplementary figures and images for: Sustainable energy policies from a complexity perspective
Source: Front Big Data. 2023 May 5;6:1114796. doi: 10.3389/fdata.2023.1114796 (PMC10204768; doi:10.3389/fdata.2023.1114796)

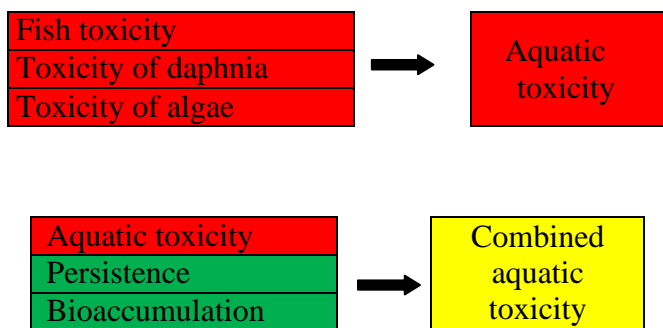

Figure 2. Risk indicator for the combined aquatic toxicity for Cu

Supplement: Supplementary file 2 [file Data_Sheet_2.pdf]
